# Cardiovascular Risk Associated With Social Determinants of Health at Individual and Area Levels

**DOI:** 10.1001/jamanetworkopen.2024.8584

**Published:** 2024-04-26

**Authors:** Mengying Xia, Jaejin An, Monika M. Safford, Lisandro D. Colantonio, Mario Sims, Kristi Reynolds, Andrew E. Moran, Yiyi Zhang

**Affiliations:** 1 Division of General Medicine, Columbia University Irving Medical Center, New York, New York; 2 Department of Research & Evaluation, Kaiser Permanente Southern California, Pasadena; 3 Department of Health Systems Science, Kaiser Permanente Bernard J. Tyson School of Medicine, Pasadena, California; 4 Division of General Internal Medicine, Department of Medicine, Weill Cornell Medicine, New York, New York; 5 Department of Epidemiology, University of Alabama at Birmingham, Birmingham; 6 Department of Social Medicine, Population, and Public Health, University of California, Riverside

## Abstract

**Importance:**

The benefit of adding social determinants of health (SDOH) when estimating atherosclerotic cardiovascular disease (ASCVD) risk is unclear.

**Objective:**

To examine the association of SDOH at both individual and area levels with ASCVD risks, and to assess if adding individual- and area-level SDOH to the pooled cohort equations (PCEs) or the Predicting Risk of CVD Events (PREVENT) equations improves the accuracy of risk estimates.

**Design, Setting, and Participants:**

This cohort study included participants data from 4 large US cohort studies. Eligible participants were aged 40 to 79 years without a history of ASCVD. Baseline data were collected from 1995 to 2007; median (IQR) follow-up was 13.0 (9.3-15.0) years. Data were analyzed from September 2023 to February 2024.

**Exposures:**

Individual- and area-level education, income, and employment status.

**Main outcomes and measures:**

ASCVD was defined as the composite outcome of nonfatal myocardial infarction, death from coronary heart disease, and fatal or nonfatal stroke.

**Results:**

A total of 26 316 participants were included (mean [SD] age, 61.0 [9.1] years; 15 494 women [58.9%]; 11 365 Black [43.2%], 703 Chinese American [2.7%], 1278 Hispanic [4.9%], and 12 970 White [49.3%]); 11 764 individuals (44.7%) had at least 1 adverse individual-level SDOH and 10 908 (41.5%) had at least 1 adverse area-level SDOH. A total of 2673 ASCVD events occurred during follow-up. SDOH were associated with increased risk of ASCVD at both the individual and area levels, including for low education (individual: hazard ratio [HR], 1.39 [95% CI, 1.25-1.55]; area: HR, 1.31 [95% CI, 1.20-1.42]), low income (individual: 1.35 [95% CI, 1.25-1.47]; area: HR, 1.28 [95% CI, 1.17-1.40]), and unemployment (individual: HR, 1.61 [95% CI, 1.24-2.10]; area: HR, 1.25 [95% CI, 1.14-1.37]). Adding area-level SDOH alone to the PCEs did not change model discrimination but modestly improved calibration. Furthermore, adding both individual- and area-level SDOH to the PCEs led to a modest improvement in both discrimination and calibration in non-Hispanic Black individuals (change in C index, 0.0051 [95% CI, 0.0011 to 0.0126]; change in scaled integrated Brier score [IBS], 0.396% [95% CI, 0.221% to 0.802%]), and improvement in calibration in White individuals (change in scaled IBS, 0.274% [95% CI, 0.095% to 0.665%]). Adding individual-level SDOH to the PREVENT plus area-level social deprivation index (SDI) equations did not improve discrimination but modestly improved calibration in White participants (change in scaled IBS, 0.182% [95% CI, 0.040% to 0.496%]), Black participants (0.187% [95% CI, 0.039% to 0.501%]), and women (0.289% [95% CI, 0.115% to 0.574%]).

**Conclusions and Relevance:**

In this cohort study, both individual- and area-level SDOH were associated with ASCVD risk; adding both individual- and area-level SDOH to the PCEs modestly improved discrimination and calibration for estimating ASCVD risk for Black individuals, and adding individual-level SDOH to PREVENT plus SDI also modestly improved calibration. These findings suggest that both individual- and area-level SDOH may be considered in future development of ASCVD risk assessment tools, particularly among Black individuals.

## Key Points

**Question:**

Does adding individual- or area-level social determinants of health (SDOH) to the pooled cohort equations (PCEs) or the Predicting Risk of CVD Events (PREVENT) equations improve accuracy of risk estimates for atherosclerotic cardiovascular disease (ASCVD)?

**Findings:**

In this cohort study of 26 316 participants from 4 large US studies, both individual- and area-level of low education, low income, and unemployment were associated with an increased risk of incident ASCVD events. Adding area-level SDOH alone to the PCEs did not improve model discrimination and modestly improved calibration, while adding both individual- and area-level SDOH to the PCEs modestly improved discrimination and calibration in non-Hispanic Black individuals; the addition of individual-level SDOH to the PREVENT plus social deprivation index (SDI) model also modestly improved calibration in non-Hispanic Black and White individuals.

**Meaning:**

The findings suggest that both individual- and area-level SDOH may be considered in future development of ASCVD risk assessment tools, particularly among Black individuals.

## Teaser

This cohort study including participants from 4 large US studies examines the association of social determinants of health—including education, income, and employment status—with improvements in common tools for assessing risk of atherosclerotic cardiovascular disease.

## Introduction

Social determinants of health (SDOH) are the conditions in the environment where people are born, live, learn, work, play, worship, and age that affect a wide range of health, functioning, and quality of life outcomes and risks.^1^ SDOH are important determinants of cardiovascular health.^2,3^ Individual- and area-level SDOH may capture social factors in different ways and represent distinct influences on cardiovascular outcomes.^4^ For example, individual-level SDOH can capture granular data specific to each individual. However, these data may be subject to social desirability bias if collected by self-report, where individuals may provide socially desirable responses rather than truthful ones, and may also be harder to collect due to their sensitive nature.^5,6^ Area-level SDOH are derived from geographic areas and capture characteristics of the neighborhood and built environment.^7^ They are more objective and can be easily collected and incorporated into a health care system setting when linked with census tract-level data.^8,9,10^ Socioeconomic status including education, household income, and employment status are the most commonly collected and studied SDOH.^4,11^ Previous studies have shown that both individual- and area-level socioeconomic status are associated with atherosclerotic cardiovascular disease (ASCVD).^12,13,14,15^

Despite the importance of SDOH in determining ASCVD risk, they are not included in the pooled cohort equations (PCEs) when estimating 10-year ASCVD risk.^13,16^ Studies have shown that PCEs systematically underestimate ASCVD risk for individuals with low socioeconomic status.^17,18^ However, it is unclear if adding either or both individual- and area-level SDOH to the PCEs improves ASCVD risk estimates. Additionally, although area-level social deprivation index (SDI) was considered as an optional variable in the recently developed American Heart Association’s Predicting Risk of CVD Events (PREVENT) equations, the role of individual-level SDOH was not assessed in PREVENT.^19^ This study sought to (1) examine the association of SDOH drawn from the education, income, and employment status at both individual and area levels with the risk of incident ASCVD events, and (2) assess if adding individual- and area-level SDOH to the PCEs or to PREVENT plus SDI improves the accuracy of ASCVD risk estimation.

## Methods

### Study Design and Cohorts

This study analyzed data from 4 large, population-based prospective cohort studies in the US: (1) Framingham Heart Study Offspring Cohort (FHS Offspring)^20^; (2) Jackson Heart Study (JHS)^21^; (3) Multi-Ethnic Study of Atherosclerosis (MESA)^22^; and (4) Reasons for Geographic And Racial Differences in Stroke Study (REGARDS).^23^ The design of each study is reported in the eMethods in Supplement 1. All study protocols were approved by the institutional review boards at participating institutions, and this study was approved by the Columbia University institutional review board. All participants provided written informed consent. The current analysis included participants aged 40 to 79 years in whom the PCEs were applicable.^24^ We excluded participants with a history of ASCVD at baseline (6841 individuals), missing SDOH (8453 individuals), missing covariates included in the PCEs (1472 individuals), or missing follow-up for incident ASCVD events (297 individuals) (eFigure 1 in Supplement 1). The results are reported based on Strengthening the Reporting of Observational Studies in Epidemiology (STROBE) reporting guidelines.

### Data Collection

Demographics and traditional ASCVD risk factors were measured using standardized protocols in each study.^20,21,22,23^ Diabetes was defined as fasting blood glucose levels of 126 mg/dL or higher (to convert to millimoles per liter, multiply by 0.0555) or the use of antidiabetes medication. Information on individual-level education (less than high school, high school or higher education), income (less than $35 000, $35 000 or higher), employment status (unemployed, not unemployed including those who were retired), and race and ethnicity (Chinese American, Hispanic, non-Hispanic Black, non-Hispanic White) were self-reported by the participants using fixed categories drawn from the source studies. Area-level SDOH data were derived by linking each participants’ residential addresses at baseline via geocoding to US census tract 2000 data at the census tract level.^25,26,27^ Specifically, we examined 3 area-level SDOH in the current analysis: (1) neighborhood education (percentage of residents aged 25 years or older with less than high school education), (2) neighborhood income (percentage of residents whose family income was below the federal poverty level), and (3) neighborhood unemployment (percentage of residents aged 16 years or older in the labor force who were unemployed). Low neighborhood income was defined as having 25% or more residents living below poverty level, as was done in a previous study.^28^ Because there were no commonly used cutoffs for area-level education and unemployment, we used the upper quartile to define low education (ie, 33% or more residents with less than high school education) and high unemployment (11% or more residents unemployed).

### Follow-Up and ASCVD Events

The primary outcome was time to the first incident ASCVD event. ASCVD events were defined as the composite outcome of nonfatal myocardial infarction (MI), death from coronary heart disease (CHD), and fatal or nonfatal stroke. Events were ascertained and adjudicated using the specific protocol of each cohort (eMethods in Supplement 1).^20,21,22,23,29^

### Statistical Analysis

Participant characteristics at the baseline visit were described for the overall pooled cohort, by individual studies, and by number of SDOH. The agreement between individual-level and area-level SDOH was assessed by Cohen κ statistics, which range between poor-to-fair agreement (below 0.40), moderate agreement (0.41 to 0.60), substantial agreement (0.61 to 0.80), and excellent agreement (0.81 to 1.00).^30,31^

To examine the association between each individual- and area-level SDOH measure with incident ASCVD events, we used 3 Cox proportional hazards models with progressive adjustment for potential confounders. The base model was adjusted for sex and age at the baseline visit. The second model was further adjusted for race and ethnicity. In a third model, we further included covariates used in the PCEs (including smoking status, total cholesterol, high-density lipoprotein cholesterol, systolic blood pressure, use of antihypertension medication, and diabetes status) and use of lipid-lowering medication to assess if the associations between SDOH and ASCVD may be explained by these traditional ASCVD risk factors. All models were stratified by study cohort to account for potential cohort effect by allowing the baseline hazard function to vary across different cohorts. The proportional hazards assumption was checked by log(–log(survival)) vs log (survival time) plots and Schoenfeld residuals. In secondary analyses, we examined the association between the numbers of adverse SDOH and ASCVD, as well as included all individual- and area-level SDOH of interest simultaneously in the same Cox model. Since all Cohen κ results between individual- and area-level SDOH were below 0.62, collinearity was not an issue. Additionally, because previous studies showed that the association between SDOH and health outcomes may differ by sex and race,^32,33^ we performed stratified analysis by sex and by race and ethnicity (Chinese American, Hispanic, non-Hispanic Black, and non-Hispanic White).

To assess if adding individual- and area-level SDOH to the PCEs improve the accuracy of ASCVD risk estimates, we fitted 4 separate Cox models: (1) a model only including the 10-year ASCVD risk estimated by the PCEs, (2) a model including the 10-year risk estimated by the PCEs plus individual-level SDOH measures, (3) a model including the 10-year risk estimated by the PCEs plus area-level SDOH measures, and (4) a model including the 10-year risk estimated by the PCEs plus both individual- and area-level SDOH measures. We assessed model discrimination by calculating Harrell C index at 10 years^34^ and assessed model calibration by using calibration plots as well as calculating scaled integrated Brier score (scaled IBS, which takes values between 0% to 100% with a higher value representing better calibration).^35,36^ We examined the changes in model performance in the overall population as well as by sex and by racial and ethnic groups. We used nonparametric bootstrapping with 500 iterations to calculate the 95% CIs for the change in Harrell C index and scaled IBS.

Additionally, although area-level SDI was considered as an optional variable in the recently developed PREVENT equations, the role of individual-level SDOH was not assessed in PREVENT.^19^ Therefore, as a secondary analysis, we assessed if adding individual-level SDOH to the PREVENT plus SDI equations may further improve estimate accuracy. In the original PREVENT plus SDI risk model, SDI was calculated at the zip code level and was not available in the current study population.^19^ Therefore, we replaced SDI with a similar area-level deprivation index developed by the Agency for Healthcare Research and Quality, which was based on 7 indicators of poverty, education, employment, and physical environment at the census tract level when calculating 10-year ASCVD risk estimated by the PREVENT plus SDI equations.^37,38^

Lastly, because approximately 20% of participants were missing information on SDOH, possibly due to unwillingness to report this information, we performed sensitivity analyses by creating missing indicator variables for these participants and including them in all the analyses (34 023 individuals). The threshold for statistical significance was a 2-sided *P* < .05. All analyses were performed with R version 4.0.2 (R Project for Statistical Computing).

## Results

A total of 26 316 participants were included in this study (mean [SD] age at baseline, 61.0 [9.1] years); 15 494 were women (58.9%) and 703 self-identified as Chinese American (2.7%), 1278 as Hispanic (4.9%), 11 365 as non-Hispanic Black (43.2%), and 12 970 as non-Hispanic White (49.3%) (Table). For individual-level SDOH, 2882 participants (11.0%) had less than high school education, 11 107 (42.2%) had low household income, and 568 (2.2%) were unemployed. For area-level SDOH, the median (IQR) for percentage of neighborhood with less than high school education was 21.8% (10.9%-32.5%), median percentage of neighborhood living below federal poverty line was 14.2% (7.0%-25.5%), and median neighborhood unemployment was 7.1% (4.3%-11.3%). Individuals with at least 1 adverse individual- or area-level SDOH were more likely to be non-Hispanic Black or to have a worse cardiovascular risk factor profile (eTables 1 and 2 in Supplement 1). The Cohen κ statistics between individual-level and area-level SDOH were 0.18 (95% CI, 0.17-0.20) for education, 0.21 (95% CI, 0.20-0.22) for income, and 0.01 (95% CI, 0.00-0.02) for unemployment (eTable 3 in Supplement 1).

**Table.** Baseline Characteristics of Study Participants

Participant characteristics	Participants, No. (%)				
	Overall (N = 26 316)	FHS Offspring (n = 1181)	JHS (n = 3443)	MESA (n = 5906)	REGARDS (n = 15 786)
Age, mean (SD), y	61.0 (9.1)	58.3 (9.0)	56.0 (10.2)	61.1 (9.5)	62.3 (8.2)
Follow-up time, median (IQR), y	13.0 (9.3-15.0)	21.9 (15.8-23.3)	13.7 (10.4-14.6)	16.8 (13.3-17.5)	12.0 (7.5-13.8)
Gender					
Women	15 494 (58.9)	620 (52.5)	2229 (64.7)	3090 (52.3)	9555 (60.5)
Men	10 822 (41.1)	561 (47.5)	1214 (35.3)	2816 (47.7)	6231 (39.5)
Race and ethnicity					
Chinese American	703 (2.7)	0	0	703 (11.9)	0
Hispanic	1278 (4.9)	0	0	1278 (21.6)	0
Non-Hispanic Black	11 365 (43.2)	0	3443 (100)	1611 (27.3)	6311 (40.0)
Non-Hispanic White	12 970 (49.3)	1181 (100)	0	2314 (39.2)	9475 (60.0)
Smoking status					
Never	13 507 (51.3)	406 (34.4)	2396 (69.6)	2953 (50.0)	7752 (49.1)
Former	9305 (35.4)	574 (48.6)	648 (18.8)	2183 (37.0)	5900 (37.4)
Current	3504 (13.3)	201 (17.0)	399 (11.6)	770 (13.0)	2134 (13.5)
BMI, mean (SD)	29.5 (6.2)	27.9 (5.0)	31.7 (7.0)	28.4 (5.4)	29.5 (6.3)
Lipids, mean (SD), mg/dL					
Total cholesterol	196.0 (38.1)	205.2 (37.6)	200.8 (38.9)	194.2 (35.3)	195.0 (38.7)
HDL cholesterol	52.7 (15.9)	51.0 (15.7)	52.4 (14.6)	51.0 (14.7)	53.6 (16.6)
LDL cholesterol	118.4 (33.9)	127.3 (33.6)	127.4 (35.8)	117.4 (31.2)	116.1 (34.0)
Triglycerides	126.2 (83.1)	134.6 (77.1)	106.9 (80.5)	131.2 (87.4)	128.0 (81.9)
Blood pressure, mean (SD), mm Hg					
Systolic	125.9 (17.2)	128.5 (18.8)	127.6 (16.3)	125.4 (20.8)	125.5 (15.8)
Diastolic	75.4 (9.7)	75.8 (9.6)	76.2 (8.7)	71.9 (10.2)	76.5 (9.3)
Diabetes	4579 (17.4)	99 (8.4)	547 (15.9)	713 (12.1)	3220 (20.4)
Use of antihypertensive medication	11 772 (44.7)	298 (25.2)	1799 (52.3)	2115 (35.8)	7560 (47.9)
Use of lipid-lowering medication	6058 (23.0)	128 (10.8)	449 (13.0)	952 (16.1)	4529 (28.7)
**SDOH characteristics**					
Individual-level SDOH					
Less than high school education	2882 (11.0)	55 (4.7)	539 (15.7)	969 (16.4)	1319 (8.4)
Annual household income <$35 000	11 107 (42.2)	480 (40.6)	1636 (47.5)	2511 (42.5)	6480 (41.0)
Unemployed	568 (2.2)	16 (1.4)	7 (0.2)	131 (2.2)	414 (2.6)
No. of adverse individual-level SDOH					
0	14 552 (55.3)	684 (57.9)	1729 (50.2)	3199 (54.2)	8940 (56.6)
1	9030 (34.3)	444 (37.6)	1251 (36.3)	1828 (31.0)	5507 (34.9)
2	2675 (10.2)	52(4.4)	458 (13.3)	854 (14.5)	1311 (8.3)
3	59 (0.2)	1 (0.1)	5 (0.1)	25 (0.4)	28 (0.2)
Area-level SDOH, median (IQR), %					
Neighborhood with less than high school education	21.8 (10.9-32.5)	5.3 (3.7-9.5)	25.5 (12.9-36.9)	20.5 (10.3-33.1)	22.3 (12.6-32.0)
Neighborhood living below federal poverty line	14.2 (7.0-25.5)	3.9 (2.4-8.4)	22.2 (11.5-34.7)	13.0 (6.4-22.3)	14.3 (7.2-24.8)
≥25% living below federal poverty line, No. (%)	6810 (25.9)	2 (0.2)	1692 (49.1)	1233 (20.9)	3883 (24.6)
Neighborhood unemployment rate	7.1 (4.3-11.3)	15.1 (13.3-19.4)	9.0 (5.5-12.1)	6.1 (3.8-10.2)	6.6 (3.9-10.3)
No. of adverse area-level SDOH^a^					
0	15 408 (58.6)	155 (13.1)	1450 (42.1)	3839 (65.0)	9964 (63.1)
1	4937 (18.8)	998 (84.5)	747 (21.7)	875 (14.8)	2317 (14.7)
2	2658 (10.1)	27 (2.3)	407 (11.8)	485 (8.2)	1739 (11.0)
3	3313 (12.6)	1 (0.1)	839 (24.4)	707 (12.0)	1766 (11.2)

Abbreviations: BMI, body mass index (calculated as weight in kilograms divided by height in meters squared); FHS Offspring, Framingham Heart Study Offspring Cohort; HDL, high-density lipoprotein; JHS, Jackson Heart Study; LDL, low-density lipoprotein; MESA, Multi-Ethnic Study of Atherosclerosis; REGARDS, Reasons for Geographic and Racial Differences in Stroke Study; SDOH, social determinants of health.

SI conversion factor: To convert total, HDL, and LDL cholesterol to millimoles per liter, multiply by 0.0259; triglycerides to millimoles per liter, 0.0113.

^a^ Adverse area-level SDOH include (1) neighborhood with less than high school education in the upper quartile (33% or higher), (2) neighborhood living below federal poverty line 25% or higher, and (3) neighborhood with unemployment rate in the upper quartile (11% or higher).

**Figure 1.**
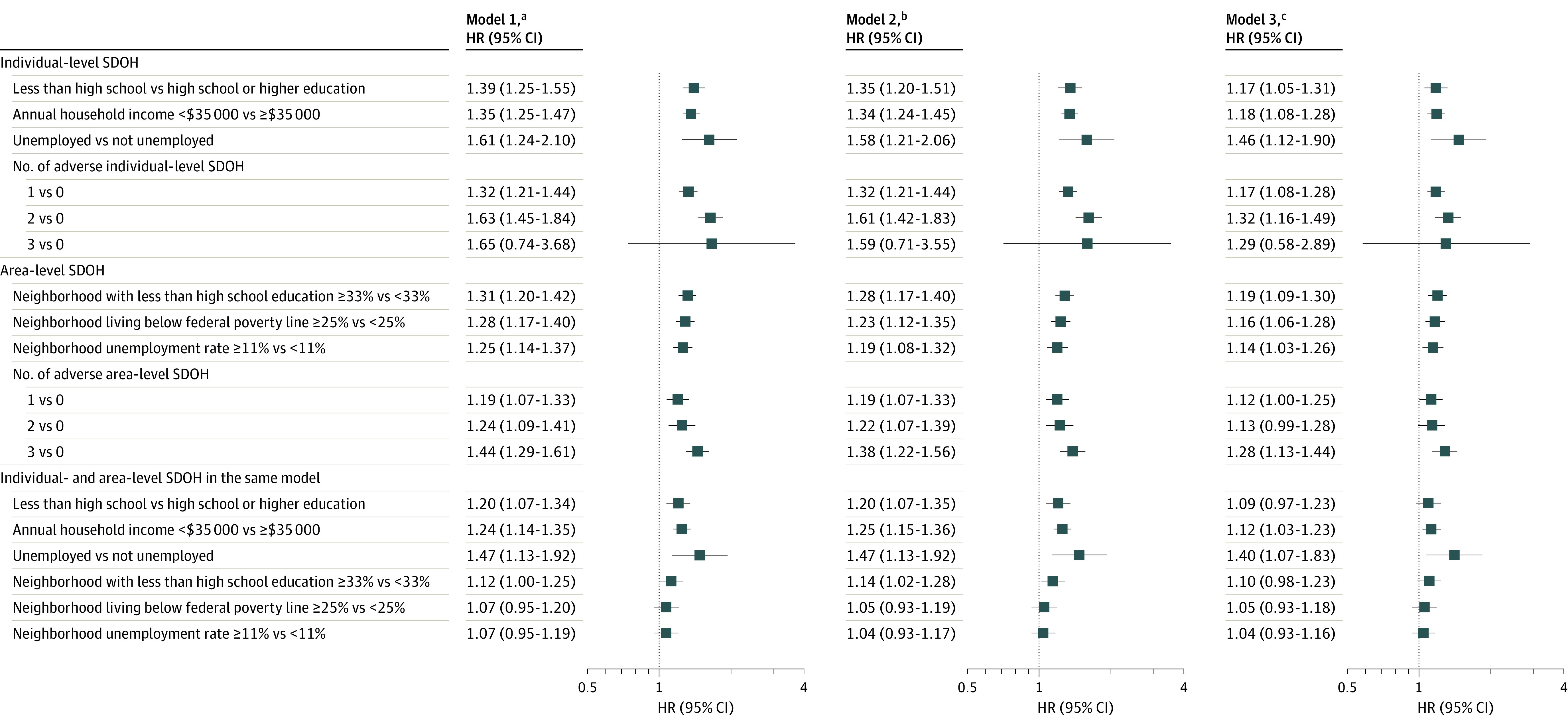
Associations of Individual-Level and Area-Level Social Determinants of Health (SDOH) With Atherosclerotic Cardiovascular Disease (ASCVD) The associations between SDOH and ASCVD were assessed using Cox proportional hazards models. All models were stratified by study cohort, allowing the baseline hazard function to vary across different cohorts. ^a^Model 1 was adjusted for sex and age at the baseline visit. ^b^Model 2 included adjustments in model 1 and was further adjusted for race and ethnicity. ^c^Model 3 included adjustments of the other models and was further adjusted for traditional ASCVD risk factors included in the pooled cohort equations (including smoking status, total cholesterol, high-density lipoprotein cholesterol, systolic blood pressure, use of antihypertension medication, and diabetes status) and use of lipid-lowering medication.

During a median (IQR) follow-up of 13.0 years (9.3-15.0 years), a total of 2673 incident ASCVD events occurred. When adjusted for age and sex, the hazard ratios (HRs) for ASCVD associated with individual-level SDOH were 1.39 (95% CI, 1.25-1.55) for less than high school education, 1.35 (95% CI, 1.25-1.47) for annual household income below $35 000, and 1.61 (95% CI, 1.24-2.10) for unemployment (Figure 1). The corresponding HRs associated with area-level SDOH were 1.31 (95% CI, 1.20-1.42) for neighborhoods with 33% or more residents with less than a high school education, 1.28 (95% CI, 1.17-1.40) for neighborhoods with 25% or more of residents living below the federal poverty line, and 1.25 (95% CI, 1.14-1.37) for neighborhoods with an unemployment rate of 11% or higher. When further adjusted for race and ethnicity, the association between SDOH and ASCVD remained the same, but results were attenuated when further controlling for traditional ASCVD risk factors in PCEs. When the numbers of adverse SDOH were examined, the magnitude of the effect size increased with the number of adverse individual- and area-level SDOH. For example, compared with participants with no adverse individual-level SDOH, the HRs associated with having 1 and 2 adverse individual-level SDOH were 1.32 (95% CI, 1.21-1.44) and 1.63 (95% CI, 1.45-1.84), respectively. Results for individuals with 3 adverse SDOH were not significant (HR, 1.65; 95% CI, 0.74-3.68). When including individual- and area-level SDOH simultaneously in the same model, all 3 individual-level SDOH and area-level education were associated with ASCVD.

In the analyses stratified by sex, we found similar associations between SDOH and ASCVD in men and women, except that effect sizes for the association between annual household income and ASCVD were higher in women (HR, 1.48; 95% CI, 1.31-1.66) than in men (HR, 1.23; 95% CI, 1.10-1.37; *P* for interaction = .006) (eTable 4 in Supplement 1). When stratified by race and ethnicity, we found similar effect sizes for the association between individual- and area-level SDOH with ASCVD across all races, except for individual- and area-level education, which had larger effect sizes for ASCVD in non-Hispanic White individuals than Hispanic individuals (eTable 5 in Supplement 1).

**Figure 2.**
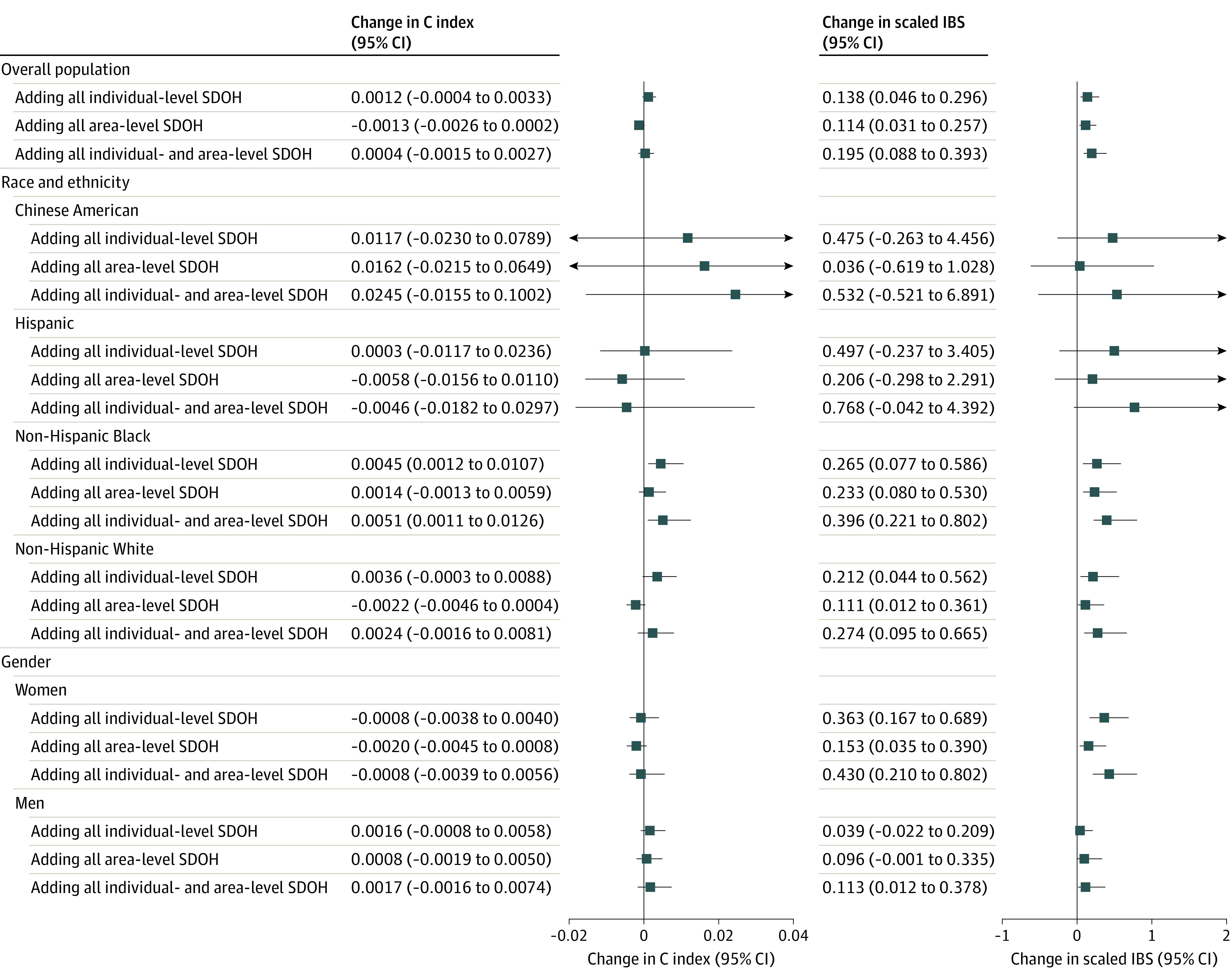
Changes in Harrell C Index and Scaled Integrated Brier Score (IBS) When Adding Individual-Level and Area-Level Social Determinants of Health (SDOH) to the Pooled Cohort Equations The 95% CI of change in C index and change in scaled IBS were calculated by nonparametric bootstrapping.

**Figure 3.**
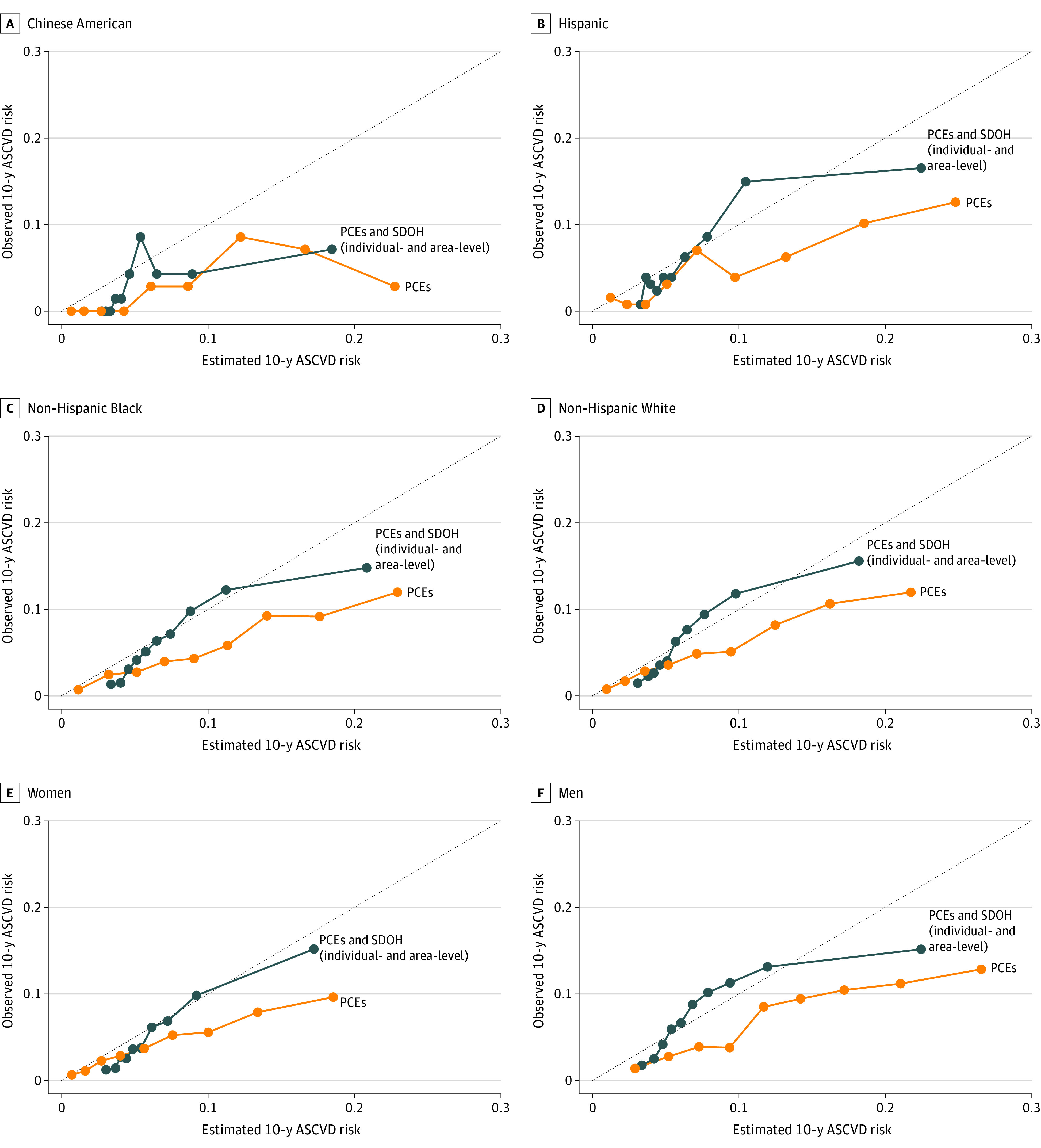
Calibration Plots of Pooled Cohort Equations (PCEs) and PCEs Plus Social Determinants of Health (SDOH) at Both Individual- and Area-Level by Race and Ethnicity and by Sex Calibration plots compared estimated with observed 10-year atherosclerotic cardiovascular disease (ASCVD) risk by decile of the estimated risk. Orange lines represent calibration curves for models with only the PCEs; blue lines, calibration curves for models with PCEs plus individual- and area-level SDOH.

Adding area-level SDOH alone to the PCEs did not improve model discrimination but modestly improved calibration (change in scaled IBS in the overall population, 0.114%; 95% CI, 0.031% to 0.257%) (Figure 2). Further adding both individual- and area-level SDOH to the PCEs led to a modest improvement in both model discrimination (change in C index, 0.0051; 95% CI, 0.0011 to 0.0126) and calibration (change in scaled IBS, 0.396%; 95% CI, 0.221% to 0.802%) in non-Hispanic Black individuals, as well as improved calibration in non-Hispanic White individuals (change in scaled IBS, 0.274%; 95% CI, 0.095% to 0.665%), women (0.430%; 95% CI, 0.210% to 0.802%), and men (0.113%; 95% CI, 0.012% to 0.378%). The calibration plot also suggested that calibration was improved in both men and women, as well as in non-Hispanic White, non-Hispanic Black, and Hispanic individuals after adding both individual- and area-level SDOH to the PCEs (Figure 3). Additionally, adding individual-level SDOH to the PREVENT plus SDI risk model did not improve model discrimination, but modestly improved calibration in non-Hispanic White (change in scaled IBS, 0.182%; 95% CI, 0.040% to 0.496%), non-Hispanic Black (0.187%; 95% CI, 0.039% to 0.501%), and in women (0.289%; 95% CI, 0.115% to 0.574%) (eFigure 2 in Supplement 1).

In sensitivity analyses not excluding participants with missing individual- or area-level SDOH, those with missing SDOH were more likely to be older, men, and non-Hispanic White and to have a worse cardiovascular risk factor profile (eTable 6 in Supplement 1). The associations between adverse individual- and area-level SDOH with incident ASCVD events were similar to those in the main analysis (eFigure 3 in Supplement 1). Additionally, missing individual-level income, individual-level employment status, or area-level SDOH in participants were associated with an increased risk of ASCVD. Adding area-level SDOH alone to the PCEs did not improve model discrimination but improved model calibration in all sex and race groups except for Chinese American (eFigure 4 in Supplement 1). Further adding both individual- and area-level SDOH to the PCEs led to a greater improvement in model discrimination in men, non-Hispanic White, and non-Hispanic Black individuals, as well as improved calibration in all sex and race groups.

## Discussion

In this analysis of over 26 000 adults from 4 large US prospective cohort studies, we found that low education, low income, and unemployment at both individual and area levels were associated with an increased risk of incident ASCVD events. Area-level education remained associated with ASCVD even after adjusting for individual-level measures. Adding area-level SDOH alone to the PCEs did not improve model discrimination but modestly improved calibration. Further adding both individual- and area-level SDOH to the PCEs led to a modest improvement in both discrimination and calibration in non-Hispanic Black individuals. Additionally, adding individual-level SDOH to PREVENT plus SDI modestly improved calibration in non-Hispanic Black and non-Hispanic White individuals. These findings suggest that both individual- and area-level SDOH may be considered in the development of future ASCVD risk assessment tools, particularly among non-Hispanic Black individuals.

Adverse SDOH are important cardiovascular risk factors and may confer ASCVD risk that is equivalent to or greater than traditional risk factors.^13,16^ Education, income, and employment status are commonly collected individual-level SDOH and have been consistently shown to be associated with ASCVD risks.^12,16,39,40,41^ Similarly, neighborhood-level SDOH were also associated with ASCVD risk.^4,12,15,42,43^ In a study of 77 101 individuals with a history of ASCVD from Kaiser Permanente Southern California, individuals living in neighborhoods with lower education and lower household income were associated with a higher risk of recurrent ASCVD events.^15^ A Swedish study of 336 295 men and 334 057 women showed that individuals from neighborhoods with higher unemployment rates were associated with a greater CHD risk.^42^

In past studies, area-level SDOH were sometimes used to approximate individual-level SDOH when individual-level data were not available. However, individual- and area-level SDOH may capture ASCVD risk differently.^4,6,16,44,45,46,47,48^ In a study of over 3 470 000 participants in the Mortality Disparities in American Communities study, there was moderate agreement among binary indicators of education, income, and employment status across individual, census tract, and county levels, with increased precision for census tract compared with county-level measures when approximating individual-level values.^47^ The SDOH-mortality associations were also found to be systematically underestimated when area-level SDOH were used as proxies for individual-level measures.^47^ Similarly, an analysis of over 97 025 individuals from the Canadian Community Health Survey found both individual- and area-level income measures were associated with premature mortality, with low agreement between individual- and area-level income measures and effect sizes that were larger for the mortality associations with individual-level measures.^49^ Consistent with previous studies, the current analysis also found low agreement between individual- and area-level SDOH of education, income, and employment status. Our study also extended those previous reports by examining the association between SDOH and ASCVD outcomes, and found that both individual- and area-level education, income, and unemployment were associated with ASCVD risk. Furthermore, area-level education remained associated with ASCVD even after adjusting for individual-level measures.

The current study found overall similar associations between individual- and area-level SDOH with ASCVD in men and women, except that individual-level household income appeared to be a stronger variable for estimating risk of ASCVD in women than in men. A 2017 meta-analysis^32^ of 44 studies with over 22 million individuals found that while SDOH were associated with CHD risk in both sexes, adverse SDOH including lower educational attainment, lower income, and higher area deprivation were associated with a significantly greater excess CHD risk in women compared with men. Additionally, our study found similar associations between individual-and area-level SDOH with ASCVD risk in non-Black and Black individuals. However, a previous study^33^ of over 25 000 individuals from a nationally representative survey of US adults found a more pronounced effect in results for the association between individual-level education and CVD risk in non-Hispanic White individuals than in non-Hispanic Black individuals. Some previous reports have proposed the hypothesis of marginalization-related diminished returns, which refers to the weaker health effects of SDOH, particularly education and income, for members of socially marginalized groups (eg, Black and Hispanic) compared with socially privileged groups (eg, non-Hispanic White).^33,50,51,52^ Findings from the current analysis need to be validated in future studies of diverse racial groups.

Previous studies have shown that the PCEs may overestimate or underestimate 10-year ASCVD risks to various degrees depending on an individual’s socioeconomic status, and adding SDOH measures to risk assessment may improve prediction accuracy.^18,53,54^ A study of more than 11 000 participants from the Atherosclerosis Risk in Communities Study found that socioeconomic status modified the association between the PCEs-estimated risk and absolute risk of ASCVD, and adding individual-level education and measures of neighborhood deprivation to the PCEs improved overall model fit.^14^ The current analysis found that adding area-level SDOH alone to the PCEs did not improve model discrimination, while further adding both individual- and area-level SDOH to the PCEs led to a modest improvement in both discrimination and calibration in non-Hispanic Black individuals. These findings suggest that both individual- and area-level SDOH may be considered in future ASCVD risk assessment tools, particularly among black individuals.

Additionally, while the recently developed PREVENT equations considered area-level SDOH as an optional predictor, the current study suggests that adding individual-level SDOH may further improve model calibration.^19^ Future studies should evaluate the benefits of considering both individual- and area-level SDOH in ASCVD risk prediction across large diverse sociodemographic groups.

### Strengths and Limitations

This study has several strengths. The primary strength lies in the unique study design, which pooled data from 4 large prospective cohort studies with high-quality exposure and outcome assessments as well as a large sample size and long follow-up duration. This allowed us to estimate the associations more reliably between SDOH with ASCVD, and more accurately evaluate the impact of SDOH on ASCVD risk assessment.

This study also has several limitations. First, our study only examined selected individual- and area-level SDOH of education, income, and unemployment, because these are the ones most consistently associated with ASCVD outcomes and most collected in research. Future studies are needed to explore the role of other SDOH such as social support, perceived discrimination, and racism in ASCVD risk prediction. Second, in the current analysis, the majority of the participants who were missing SDOH were missing information on individual-level SDOH, likely due to the personal and sensitive nature of such information and participants’ unwillingness to report.^6,55,56^ Indeed, in sensitivity analyses not excluding those participants with missing SDOH, we found that participants with missing individual-level income or employment status were significantly associated with an increased risk of ASCVD, suggesting the mechanism of missing SDOH was likely missing not at random. Future studies should explore strategies to improve the collection of relevant SDOH history from participants and reduce the occurrence of missing information. Third, our study primarily included non-Hispanic White and non-Hispanic Black individuals, with a limited number of other racial groups. Further studies are needed to assess the association between SDOH and ASCVD in diverse populations of Hispanics, Asians, and other racial minority groups with larger sample sizes. Fourth, we did not adjust individual-level income for inflation because income was self-reported using fixed categories. This may result in some participants being misclassified into low- or high-income categories. Lastly, we reported nominal statistical associations and *P* values for all analyses as correction for multiple testing may increase the risk of type II errors.^57^ We recognize that although this approach minimizes loss of true positive findings, it may also risk identification of false associations and results from the current analysis require confirmation in other studies.

## Conclusions

Both individual- and area-level SDOH of low education, low income, and unemployment were associated with an increased risk of incident ASCVD events. Adding area-level SDOH alone to the PCEs did not improve model discrimination and modestly improved calibration, while adding both individual- and area-level SDOH to the PCEs modestly improved discrimination and calibration in non-Hispanic Black individuals. Addition of individual-level SDOH to the PREVENT plus SDI also modestly improved calibration in non-Hispanic Black and White individuals. These findings suggest that both individual- and area-level SDOH may be considered in future development of ASCVD risk assessment tools, particularly among non-Hispanic Black individuals.

## Notes

Supplement 1.
eMethods.
**eTable 1.** Baseline Characteristics Stratified by Number of Individual-Level SDOH **eTable 2.** Baseline Characteristics Stratified by Number of Area-Level SDOH **eTable 3.** Cohen Kappa (95% CI) Between Individual-Level and Area-Level SDOH **eTable 4.** Associations of Individual-Level and Area-Level Social Determinants of Health (SDOH) With Atherosclerotic Cardiovascular Disease (ASCVD), Stratified by Sex **eTable 5.** Associations of Social Determinants of Health (SDOH) With Atherosclerotic Cardiovascular Disease (ASCVD), Stratified by Race **eTable 6.** Baseline Characteristics Comparing Participants Included in the Main Analysis vs Those Who Were Excluded Due to Missing SDOH Variables **eFigure 1.** Flowchart of Study Design **eFigure 2.** Changes in Harrell C-Index and Scaled Integrated Brier Score (Scaled IBS) When Adding Individual-Level SDOH to PREVENT + SDI **eFigure 3.** Associations of Individual-Level and Area-Level Social Determinants of Health (SDOH) With Atherosclerotic Cardiovascular Disease (ASCVD), Not Excluding Participants With Missing Individual- or Area-Level SDOH **eFigure 4.** Changes in Harrell C-Index and Scaled Integrated Brier Score (Scaled IBS) When Adding Individual-Level and Area-Level SDOH to the Pooled Cohort Equations (PCEs), Not Excluding Participants With Missing Individual- or Area-Level SDOH
eReferences

Supplement 2.
Data Sharing Statement
