# Supplementary material for: Cardiovascular Risk Associated With Social Determinants of Health at Individual and Area Levels
Source: JAMA Netw Open. 2024 Apr 26;7(4):e248584. doi: 10.1001/jamanetworkopen.2024.8584 (PMC11053380; doi:10.1001/jamanetworkopen.2024.8584)
Supplement: Supplement 1. — eMethods. eTable 1. Baseline Characteristics Stratified by Number of Individual-Level SDOH eTable 2. Baseline Characteristics Stratified by Number of Area-Level SDOH eTable 3. Cohen Kappa (95% CI) Between Individual-Level and Area-Level SDOH eTable 4. Associations of Individual-Level and Area-Level Social Determinants of Health (SDOH) With Atherosclerotic Cardiovascular Disease (ASCVD), Stratified by Sex eTable 5. Associations of Social Determinants of Health (SDOH) With Atherosclerotic Cardiovascular Disease (ASCVD), Stratified by Race eTable 6. Baseline Characteristics Comparing Participants Included in the Main Analysis vs Those Who Were Excluded Due to Missing SDOH Variables eFigure 1. Flowchart of Study Design eFigure 2. Changes in Harrell C-Index and Scaled Integrated Brier Score (Scaled IBS) When Adding Individual-Level SDOH to PREVENT + SDI eFigure 3. Associations of Individual-Level and Area-Level Social Determinants of Health (SDOH) With Atherosclerotic Cardiovascular Disease (ASCVD), Not Excluding Participants With Missing Individual- or Area-Level SDOH eFigure 4. Changes in Harrell C-Index and Scaled Integrated Brier Score (Scaled IBS) When Adding Individual-Level and Area-Level SDOH to the Pooled Cohort Equations (PCEs), Not Excluding Participants With Missing Individual- or Area-Level SDOH eReferences [file jamanetwopen-e248584-s001.pdf]

Supplemental Online Content

Xia M, An J, Safford MM, et al. Cardiovascular risk associated with social determinants of health at individual and area levels. *JAMA Netw Open.* 2024;7(4):e248584. doi:10.1001/jamanetworkopen.2024.8584

eMethods

eTable 1. Baseline Characteristics Stratified by Number of Individual-Level SDOH

eTable 2. Baseline Characteristics Stratified by Number of Area-Level SDOH

eTable 3. Cohen Kappa (95% CI) Between Individual-Level and Area-Level SDOH

eTable 4. Associations of Individual-Level and Area-Level Social Determinants of Health (SDOH) With Atherosclerotic Cardiovascular Disease (ASCVD), Stratified by Sex

eTable 5. Associations of Social Determinants of Health (SDOH) With Atherosclerotic Cardiovascular Disease (ASCVD), Stratified by Race

eTable 6. Baseline Characteristics Comparing Participants Included in the Main Analysis vs Those Who Were Excluded Due to Missing SDOH Variables

eFigure 1. Flowchart of Study Design

eFigure 2. Changes in Harrell C-Index and Scaled Integrated Brier Score (Scaled IBS) When Adding Individual-Level SDOH to PREVENT + SDI

eFigure 3. Associations of Individual-Level and Area-Level Social Determinants of Health (SDOH) With Atherosclerotic Cardiovascular Disease (ASCVD), Not Excluding Participants With Missing Individual- or Area-Level SDOH

eFigure 4. Changes in Harrell C-Index and Scaled Integrated Brier Score (Scaled IBS) When Adding Individual-Level and Area-Level SDOH to the Pooled Cohort Equations (PCEs), Not Excluding Participants With Missing Individual- or Area-Level SDOH

eReferences

This supplemental material has been provided by the authors to give readers additional information about their work.

© 2024 Xia M et al. *JAMA Network Open.*

eMethods.

Atherosclerotic Cardiovascular Disease Risk Associated with Social Determinants of Health at Individual and Area Levels

Study cohorts

The Framingham Heart Study Offspring Cohort (FHS Offspring) is a prospective cohort study of 5,124 individuals 5 to 70 years of age who were offspring (or offspring's spouses) of the Original Cohort of the Framingham Heart Study.[1] The baseline visit was carried out in 1971, with 8 subsequent in-person follow-up visits. The current analysis only included visit data from exam 6 (1995-1998), exam 7 (1998-2001), and exam 8 (2005 to 2008) so that the study time period was comparable to those from the other cohorts included in the analysis.

The Jackson Heart Study (JHS) is a prospective cohort study of 5,306 African Americans aged 21 years and older, who were recruited from the Jackson, Mississippi metropolitan area to investigate the etiology of cardiovascular disease among African Americans.[2] The baseline visit was carried out during between 2000 and 2004, with 2 subsequent in-person follow-up visits.

The Multi-Ethnic Study of Atherosclerosis (MESA) Study is a multicenter prospective cohort study of 6,814 individuals 45 to 84 years of age free of clinical cardiovascular disease recruited from 6 US communities (Baltimore, Maryland; Chicago, Illinois; Forsyth County, North Carolina; Los Angeles County, California; northern Manhattan, New York; and St. Paul, Minnesota).[3] The baseline visit was carried out during 2000-2002, with 5 subsequent in-person follow-up visits.

The REasons for Geographic And Racial Differences in Stroke (REGARDS) enrolled a population-based sample of 30,239 Black and White adults aged ≥45 years from the 48 contiguous US states and the District of Columbia.[4] Sampling aimed to balance on race and sex, with oversampling of Black individuals, as well as oversampling of the Stroke Buckle (coastal North and South Carolina and Georgia) and the Stroke Belt (the remainder of North and South Carolina and Georgia, and Alabama, Mississippi, Louisiana, Arkansas, and Tennessee) regions with the highest stroke mortality in the US. The baseline visit was carried out between 2003-2007, and participants were contacted every 6 months by telephone to detect potential study outcomes.

Follow-up and ASCVD Events

The primary outcome was time to the first incident ASCVD event. ASCVD events were defined as nonfatal myocardial infarction (MI), death from coronary heart disease (CHD), and fatal or nonfatal stroke. Diagnosis of MI generally required at least two of the following: symptoms indicative of ischemia, electrocardiographic or other imaging abnormalities consistent with MI, and a rising and/or falling pattern of cardiac biomarkers over at least 6 hours with a peak above the upper limit of normal. Diagnosis of stroke generally required a persistent central neurologic deficit lasting >24 hours and/or brain imaging consistent with acute stroke.

© 2024 Xia M et al. *JAMA Network Open.*

eTable 1. Baseline characteristics stratified by number of individual-level SDOH

Participant characteristics	0 (N=14,552)	1-2 (N=11,705)	3 (N=59)
**Age, year**	59.6 (8.7)	62.8 (9.2)	58.6 (8.8)
**Follow-up time**	13.3 (10.4, 15.1)	12.7 (8.3, 14.8)	11.6 (5.4, 14.5)
**Sex**			
Female	7545 (51.8%)	7918 (67.6%)	31 (52.5%)
Male	7007 (48.2%)	3787 (32.4%)	28 (47.5%)
**Race and ethnicity**			
Non-Hispanic White	8586 (59.0%)	4375 (37.4%)	9 (15.3%)
Non-Hispanic Black	5348 (36.8%)	5991 (51.2%)	26 (44.1%)
Hispanic	351 (2.4%)	905 (7.7%)	22 (37.3%)
Chinese-American	267 (1.8%)	434 (3.7%)	2 (3.4%)
**Smoking status**			
Never	7574 (52.0%)	5904 (50.4%)	29 (49.2%)
Former	5398 (37.1%)	3891 (33.2%)	16 (27.1%)
Current	1580 (10.9%)	1910 (16.3%)	14 (23.7%)
**BMI, kg/m^2**	29.0 (5.8)	30.0 (6.7)	30.8 (6.7)
**Lipids, mg/dL**			
Total cholesterol	194.9 (36.6)	197.4 (39.7)	197.1 (36.5)
HDL cholesterol	52.5 (16.0)	53.0 (15.8)	49.7 (13.5)
LDL cholesterol	117.9 (32.7)	118.9 (35.3)	119.5 (34.8)
Triglycerides	123.8 (79.5)	129.2 (87.5)	139.5 (71.2)

© 2024 Xia M et al. *JAMA Network Open.*

**Blood pressure, mm Hg**			
Systolic	123.9 (16.3)	128.3 (18.1)	126.8 (20.5)
Diastolic	75.4 (9.4)	75.5 (10.0)	76.1 (11.1)
**Diabetes**	1929 (13.3%)	2636 (22.5%)	14 (23.7%)
**Use of antihypertensive medication**	5807 (39.9%)	5941 (50.8%)	24 (40.7%)
**Use of lipid-lowering medication**	3347 (23.0%)	2702 (23.1%)	9 (15.3%)

© 2024 Xia M et al. *JAMA Network Open.*

eTable 2. Baseline characteristics stratified by number of area-level SDOH

Participant characteristics	0 (N=15,408)	1-2 (N=7,595)	3 (N=3,313)
**Age, year**	61.1 (9.0)	60.9 (9.0)	61.0 (9.5)
**Follow-up time**	13.1 (9.6, 14.9)	13.1 (9.0, 15.3)	12.6 (8.3, 14.7)
**Sex**			
Female	8701 (56.5%)	4655 (61.3%)	2138 (64.5%)
Male	6707 (43.5%)	2940 (38.7%)	1175 (35.5%)
**Race and ethnicity**			
Non-Hispanic White	9474 (61.5%)	3104 (40.9%)	392 (11.8%)
Non-Hispanic Black	4981 (32.3%)	3844 (50.6%)	2540 (76.7%)
Hispanic	538 (3.5%)	381 (5.0%)	359 (10.8%)
Chinese-American	415 (2.7%)	266 (3.5%)	22 (0.7%)
**Smoking status**			
Never	7963 (51.7%)	3797 (50.0%)	1747 (52.7%)
Former	5688 (36.9%)	2654 (34.9%)	963 (29.1%)
Current	1757 (11.4%)	1144 (15.1%)	603 (18.2%)
**BMI, kg/m^2**	29.0 (5.9)	29.9 (6.5)	30.9 (6.8)
**Lipids, mg/dL**			
Total cholesterol	195.7 (37.5)	196.8 (38.5)	195.8 (39.4)
HDL cholesterol	53.0 (16.1)	52.1 (15.8)	52.9 (15.4)
LDL cholesterol	117.4 (33.2)	119.6 (34.4)	119.9 (36.0)
Triglycerides	128.1 (85.2)	126.7 (81.7)	116.5 (75.8)

© 2024 Xia M et al. *JAMA Network Open.*

**Blood pressure, mm Hg**			
Systolic	124.5 (16.8)	127.5 (17.6)	128.5 (17.7)
Diastolic	74.9 (9.6)	76.0 (9.7)	76.3 (9.6)
**Diabetes**	2302 (14.9%)	1490 (19.6%)	787 (23.8%)
**Use of antihypertensive medication**	6424 (41.7%)	3528 (46.5%)	1820 (54.9%)
**Use of lipid-lowering medication**	3705 (24.0%)	1641 (21.6%)	712 (21.5%)

© 2024 Xia M et al. *JAMA Network Open.*

eTable 3. Cohen Kappa (95% CI) between individual-level and area-level SDOH

	Less than high school education	Annual household income <$35,000	Unemployed	Neighborhood with less than high school education ≥33%	Neighborhood living below federal poverty line ≥25%	Neighborhood unemployment rate ≥11%
**Less than high school education**	1.00	-	-	-	-	-
**Annual household income <$35,000**	0.21<br>(0.20, 0.22)	1.00	-	-	-	-
**Unemployed**	0.00<br>(-0.01, 0.01)	0.02<br>(0.02, 0.03)	1.00	-	-	-
**Neighborhood with less than high school education ≥33%**	0.18<br>(0.17, 0.20)	0.25<br>(0.24, 0.26)	0.01<br>(0.01, 0.02)	1.00	-	-
**Neighborhood living below federal poverty line ≥25%**	0.16<br>(0.14, 0.17)	0.21<br>(0.20, 0.22)	0.01<br>(0.00, 0.01)	0.61<br>(0.60, 0.62)	1.00	-
**Neighborhood unemployment rate ≥11%**	0.11<br>(0.09, 0.12)	0.16<br>(0.15, 0.18)	0.01<br>(0.00, 0.02)	0.47<br>(0.45, 0.48)	0.40<br>(0.39, 0.41)	1.00

© 2024 Xia M et al. *JAMA Network Open.*

eTable 4. Associations of individual-level and area-level social determinants of health (SDOH) with atherosclerotic cardiovascular disease (ASCVD), stratified by sex

	Age-adjusted Hazard Ratio (95% CI)		P-value
	Female	Male	
**Individual-level SDOH**			
Less than high school vs. high school or higher education	1.50 (1.30,1.73)	1.27 (1.08,1.50)	0.086
Annual household income <$35,000 vs. ≥$35,000	1.48 (1.31,1.66)	1.23 (1.10,1.37)	0.006
Unemployed vs. not unemployed	1.43 (0.97,2.12)	1.75 (1.22,2.51)	0.239
Number of adverse individual-level SDOH 2-3 vs. 0-1	1.55 (1.34,1.79)	1.28 (1.08,1.53)	0.063
**Area-level SDOH**			
Neighborhood with less than high school education ≥33% vs. <33%	1.31 (1.16,1.47)	1.30 (1.15,1.47)	0.729
Neighborhood living below federal poverty line ≥25% vs. <25%	1.30 (1.15,1.47)	1.24 (1.09,1.41)	0.316
Neighborhood unemployment rate ≥11% vs. 11%	1.19 (1.05,1.35)	1.31 (1.15,1.50)	0.335
Number of adverse area-level SDOH 2-3 vs. 0-1	1.31 (1.16,1.48)	1.26 (1.11,1.44)	0.439
**Individual- and area-level SDOH in the same model**			
Less than high school vs. high school or higher education	1.28 (1.10,1.50)	1.11 (0.94,1.33)	0.243
Annual household income <$35,000 vs. ≥$35,000	1.35 (1.19,1.53)	1.12 (0.99,1.27)	0.013
Unemployed vs. not unemployed	1.32 (0.89,1.95)	1.61 (1.12,2.31)	0.275
Neighborhood with less than high school education ≥33% vs. <33%	1.10 (0.94,1.28)	1.15 (0.98,1.35)	0.550
Neighborhood living below federal poverty line ≥25% vs. <25%	1.12 (0.95,1.32)	1.00 (0.84,1.19)	0.246
Neighborhood unemployment rate ≥11% vs. <11%	0.98 (0.84,1.15)	1.16 (0.98,1.37)	0.134

© 2024 Xia M et al. *JAMA Network Open.*

eTable 5. Associations of social determinants of health (SDOH) with atherosclerotic cardiovascular disease (ASCVD), stratified by race

	Age- and sex-adjusted Hazard Ratio (95% CI)				P-value
	Non-Hispanic White	Non-Hispanic Black	Hispanic	Chinese-American	
**Individual-level SDOH**					
Less than high school vs. high school or higher education	1.70 (1.39, 2.08)	1.33 (1.15, 1.56)	0.95 (0.70, 1.29)	1.35 (0.75, 2.40)	0.009
Annual household income <$35,000 vs. ≥$35,000	1.34 (1.20, 1.51)	1.39 (1.23, 1.58)	1.16 (0.83, 1.63)	0.83 (0.47, 1.48)	0.430
Unemployed vs. not unemployed	1.13 (0.70, 1.83)	1.91 (1.33, 2.75)	1.58 (0.74, 3.37)	3.12 (0.74, 13.20)	0.282
Number of adverse individual-level SDOH 2-3 vs. 0-1	1.63 (1.31, 2.03)	1.37 (1.18, 1.60)	1.13 (0.83, 1.54)	1.63 (0.92, 2.88)	0.140
**Area-level SDOH**					
Neighborhood with less than high school education ≥33% vs. <33%	1.34 (1.16, 1.56)	1.32 (1.17, 1.49)	1.07 (0.79, 1.45)	0.74 (0.43, 1.28)	0.107
Neighborhood living below federal poverty line ≥25% vs. <25%	1.23 (1.04, 1.46)	1.26 (1.11, 1.42)	1.17 (0.87, 1.59)	0.96 (0.47, 1.96)	0.915
Neighborhood unemployment rate ≥11% vs. 11%	1.32 (1.08, 1.60)	1.20 (1.06, 1.35)	1.02 (0.74, 1.40)	0.71 (0.22, 2.29)	0.443
Number of adverse area-level SDOH 2-3 vs. 0-1	1.31 (1.09, 1.57)	1.26 (1.12, 1.42)	1.11 (0.82, 1.50)	0.76 (0.34, 1.68)	0.534
**Individual- and area-level SDOH in the same model**					
Less than high school vs. high school or higher education	1.50 (1.22, 1.85)	1.17 (1.00, 1.38)	0.85 (0.61, 1.19)	1.54 (0.83, 2.85)	0.020
Annual household income <$35,000 vs. ≥$35,000	1.25 (1.11, 1.41)	1.26 (1.10, 1.44)	1.20 (0.83, 1.72)	0.81 (0.44, 1.47)	0.783
Unemployed vs. not unemployed	1.04 (0.64, 1.68)	1.79 (1.24, 2.57)	1.55 (0.72, 3.35)	3.52 (0.82, 15.04)	0.205
Neighborhood with less than high school education ≥33% vs. <33%	1.21 (1.01, 1.44)	1.17 (1.00, 1.38)	0.99 (0.66, 1.49)	0.69 (0.37, 1.27)	0.235
Neighborhood living below federal poverty line ≥25% vs. <25%	0.99 (0.80, 1.23)	1.05 (0.89, 1.24)	1.36 (0.86, 2.15)	1.10 (0.50, 2.41)	0.648
Neighborhood unemployment rate ≥11% vs. 11%	1.17 (0.94, 1.46)	1.03 (0.89, 1.18)	0.81 (0.52, 1.27)	0.82 (0.23, 2.87)	0.459

© 2024 Xia M et al. *JAMA Network Open.*

eTable 6. Baseline characteristics comparing participants included in the main analysis vs those who were excluded due to missing SDOH variables

Participant characteristics	Included in the main analysis (N=26,316)	Excluded from the main analysis due to missing SDOH (N=7,707)	P-value
**Age, year**	61.0 (9.1)	62.4 (9.4)	<0.001
**Follow-up time**	12.0 (4.8)	10.5 (7.0)	<0.001
**Sex**			<0.001
Female	15494 (58.9%)	4213 (54.7%)	
Male	10822 (41.1%)	3494 (45.3%)	
**Race and ethnicity**			<0.001
Non-Hispanic White	12970 (49.3%)	4736 (61.5%)	
Non-Hispanic Black	11365 (43.2%)	2758 (35.8%)	
Hispanic	1278 (4.9%)	149 (1.9%)	
Chinese-American	703 (2.7%)	64 (0.8%)	
**Smoking status**			<0.001
Never	13507 (51.3%)	3306 (42.9%)	
Former	9305 (35.4%)	3187 (41.4%)	
Current	3504 (13.3%)	1214 (15.8%)	
**BMI, kg/m^2**	29.5 (6.2)	29.0 (6.2)	<0.001
**Lipids, mg/dL**			
Total cholesterol	196.0 (38.1)	199.1 (40.6)	<0.001
HDL cholesterol	52.7 (15.9)	52.0 (16.3)	0.001

© 2024 Xia M et al. *JAMA Network Open.*

LDL cholesterol	118.4 (33.9)	120.6 (35.0)	<0.001
Triglycerides	126.2 (83.1)	134.4 (108.2)	<0.001
**Blood pressure, mm Hg**			
Systolic	125.9 (17.2)	128.6 (18.0)	<0.001
Diastolic	75.4 (9.6)	76.5 (9.9)	<0.001
**Diabetes**	4579 (17.4%)	1500 (19.5%)	<0.001
**Use of antihypertensive medication**	11772 (44.7%)	3341 (43.4%)	0.033
**Use of lipid-lowering medication**	6058 (23.0%)	1662 (21.6%)	0.008

© 2024 Xia M et al. *JAMA Network Open.*

eFigure 1. Flowchart of study design

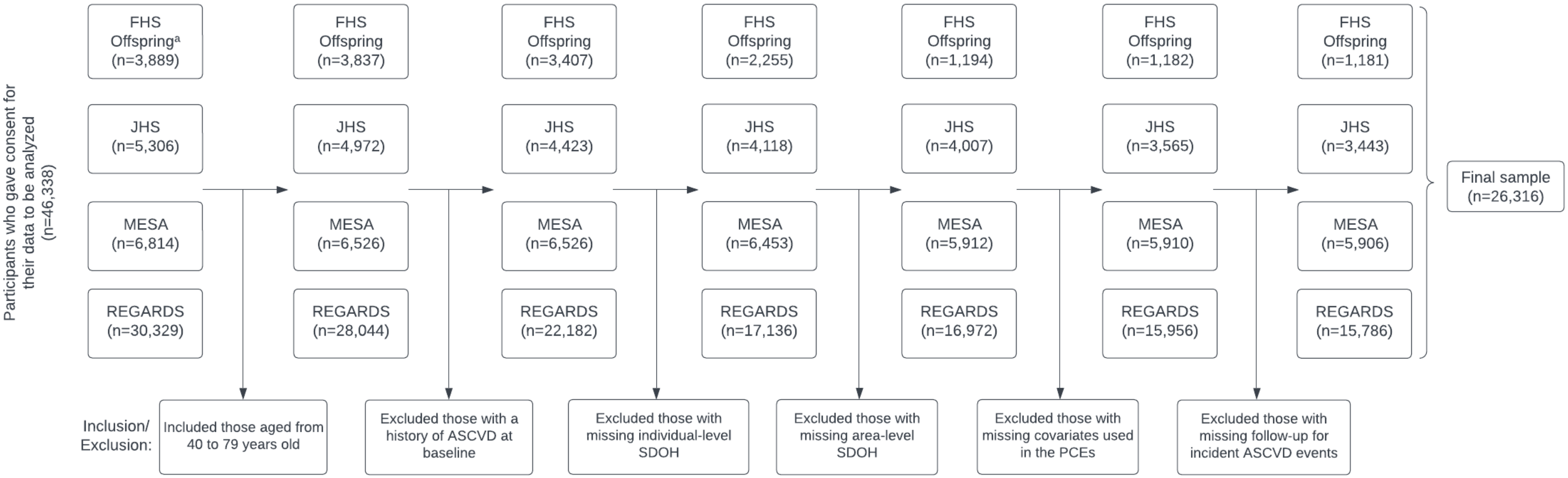

[a] In the FHS Offspring cohort, only exam 6, 7, 8 were included.

© 2024 Xia M et al. *JAMA Network Open.*

eFigure 2. Changes in Harrell C-Index and scaled Integrated Brier Score (scaled IBS) when adding individual-level SDOH to PREVENT+SDI

	Change in C-index (95% CI)	Change in scaled IBS (95% CI)
**Overall population**		
Adding all individual-level SDOH to PREVENT+SDI	-0.0007 (-0.0024, 0.0010)	0.075 (0.021, 0.194)
**Non-Hispanic White**		
Adding all individual-level SDOH to PREVENT+SDI	0.0001 (-0.0045, 0.0051)	0.182 (0.040, 0.496)
**Non-Hispanic Black**		
Adding all individual-level SDOH to PREVENT+SDI	0.0031 (-0.0001, 0.0093)	0.187 (0.039, 0.501)
**Hispanic**		
Adding all individual-level SDOH to PREVENT+SDI	0.0021 (-0.0091, 0.0245)	0.552 (-0.098, 3.363)
**Chinese-American**		
Adding all individual-level SDOH to PREVENT+SDI	0.0104 (-0.0207, 0.0622)	0.754 (-0.176, 8.040)
**Female**		
Adding all individual-level SDOH to PREVENT+SDI	-0.0077 (-0.0141, -0.0001)	0.289 (0.115, 0.574)
**Male**		
Adding all individual-level SDOH to PREVENT+SDI	0.0005 (-0.0016, 0.0047)	0.003 (-0.038, 0.158)

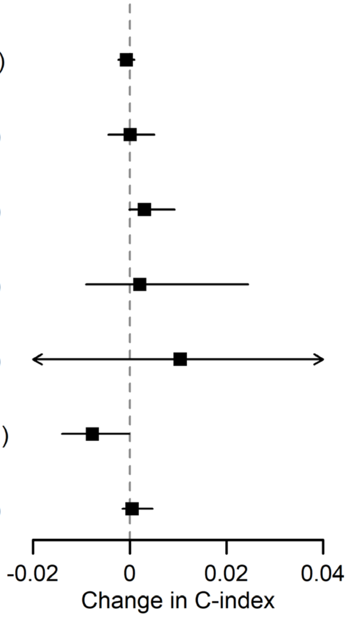

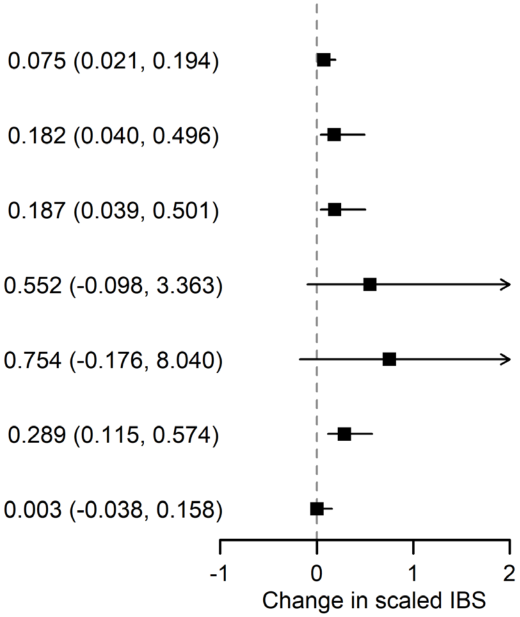

© 2024 Xia M et al. *JAMA Network Open.*

eFigure 3. Associations of individual-level and area-level social determinants of health (SDOH) with atherosclerotic cardiovascular disease (ASCVD), not excluding participants with missing individual- or area-level SDOH
Same model adjustments as in the main analysis.

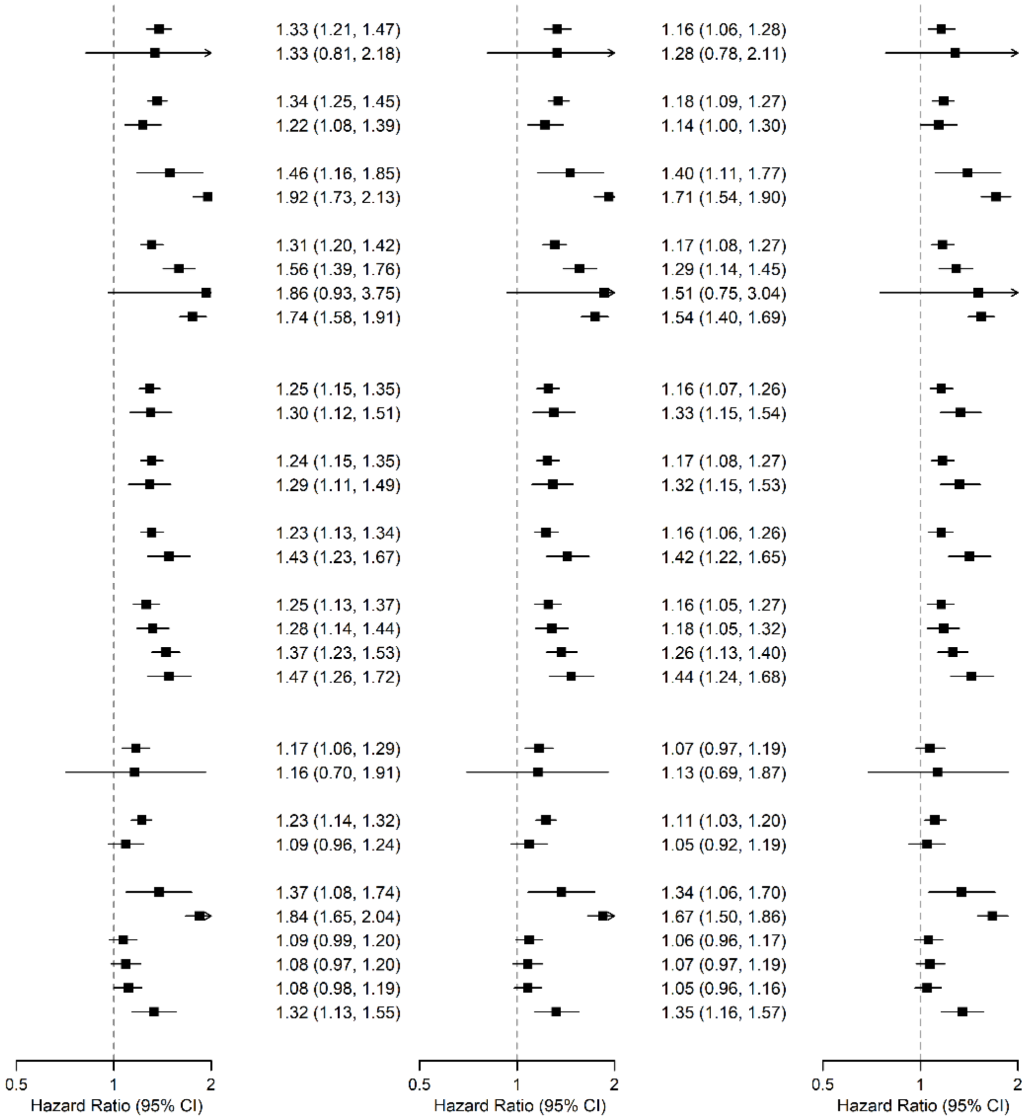

	Model1[a]	Model2[b]	Model3[c]
**Individual-level SDOH**			
Education			
Less than high school vs. high school or higher education	1.38 (1.26, 1.51)	1.33 (1.21, 1.47)	1.16 (1.06, 1.28)
Not available or not willing to report vs. high school or higher education	1.34 (0.82, 2.20)	1.33 (0.81, 2.18)	1.28 (0.78, 2.11)
Annual household income			
<$35,000 vs. ≥$35,000	1.36 (1.27, 1.46)	1.34 (1.25, 1.45)	1.18 (1.09, 1.27)
Not available or not willing to report vs. ≥$35,000	1.23 (1.08, 1.40)	1.22 (1.08, 1.39)	1.14 (1.00, 1.30)
Employment status			
Unemployed vs. not unemployed	1.49 (1.18, 1.88)	1.46 (1.16, 1.85)	1.40 (1.11, 1.77)
Not available or not willing to report vs. not unemployed	1.95 (1.76, 2.17)	1.92 (1.73, 2.13)	1.71 (1.54, 1.90)
Number of adverse individual-level SDOH			
1 vs. 0	1.31 (1.21, 1.42)	1.31 (1.20, 1.42)	1.17 (1.08, 1.27)
2 vs. 0	1.59 (1.42, 1.78)	1.56 (1.39, 1.76)	1.29 (1.14, 1.45)
3 vs. 0	1.93 (0.96, 3.86)	1.86 (0.93, 3.75)	1.51 (0.75, 3.04)
Not available or not willing to report vs. 0	1.75 (1.60, 1.93)	1.74 (1.58, 1.91)	1.54 (1.40, 1.69)
**Area-level SDOH**			
Neighborhood education			
Neighborhood with less than high school education ≥33% vs. <33%	1.29 (1.20, 1.39)	1.25 (1.15, 1.35)	1.16 (1.07, 1.26)
Not available vs. <33%	1.30 (1.12, 1.51)	1.30 (1.12, 1.51)	1.33 (1.15, 1.54)
Neighborhood poverty			
Neighborhood living below federal poverty line ≥25% vs. <25%	1.31 (1.21, 1.42)	1.24 (1.15, 1.35)	1.17 (1.08, 1.27)
Not available vs. <25%	1.29 (1.11, 1.50)	1.29 (1.11, 1.49)	1.32 (1.15, 1.53)
Neighborhood employment status			
Neighborhood unemployment rate ≥11% vs. <11%	1.31 (1.21, 1.42)	1.23 (1.13, 1.34)	1.16 (1.06, 1.26)
Not available vs. <11%	1.48 (1.27, 1.72)	1.43 (1.23, 1.67)	1.42 (1.22, 1.65)
Number of adverse area-level SDOH			
1 vs. 0	1.26 (1.15, 1.38)	1.25 (1.13, 1.37)	1.16 (1.05, 1.27)
2 vs. 0	1.32 (1.18, 1.48)	1.28 (1.14, 1.44)	1.18 (1.05, 1.32)
3 vs. 0	1.45 (1.31, 1.60)	1.37 (1.23, 1.53)	1.26 (1.13, 1.40)
Not available vs. 0	1.48 (1.27, 1.73)	1.47 (1.26, 1.72)	1.44 (1.24, 1.68)
**Individual- and area-level SDOH in the same model**			
Education			
Less than high school vs. high school or higher education	1.17 (1.06, 1.29)	1.17 (1.06, 1.29)	1.07 (0.97, 1.19)
Not available or not willing to report vs. high school or higher education	1.16 (0.71, 1.92)	1.16 (0.70, 1.91)	1.13 (0.69, 1.87)
Annual household income			
<$35,000 vs. ≥$35,000	1.22 (1.13, 1.31)	1.23 (1.14, 1.32)	1.11 (1.03, 1.20)
Not available or not willing to report vs. ≥$35,000	1.09 (0.96, 1.24)	1.09 (0.96, 1.24)	1.05 (0.92, 1.19)
Employment status			
Unemployed vs. not unemployed	1.38 (1.09, 1.74)	1.37 (1.08, 1.74)	1.34 (1.06, 1.70)
Not available or not willing to report vs. not unemployed	1.84 (1.66, 2.05)	1.84 (1.65, 2.04)	1.67 (1.50, 1.86)
Neighborhood with less than high school education ≥33% vs. <33%	1.07 (0.97, 1.18)	1.09 (0.99, 1.20)	1.06 (0.96, 1.17)
Neighborhood living below federal poverty line ≥25% vs. <25%	1.09 (0.98, 1.21)	1.08 (0.97, 1.20)	1.07 (0.97, 1.19)
Neighborhood unemployment rate ≥11% vs. <11%	1.11 (1.00, 1.22)	1.08 (0.98, 1.19)	1.05 (0.96, 1.16)
Missing neighborhood-level SDOH vs. non-missing	1.33 (1.14, 1.56)	1.32 (1.13, 1.55)	1.35 (1.16, 1.57)

© 2024 Xia M et al. *JAMA Network Open.*

eFigure 4. Changes in Harrell C-Index and scaled Integrated Brier Score (scaled IBS) when adding individual-level and area-level SDOH to the Pooled Cohort Equations (PCEs), not excluding participants with missing individual- or area-level SDOH

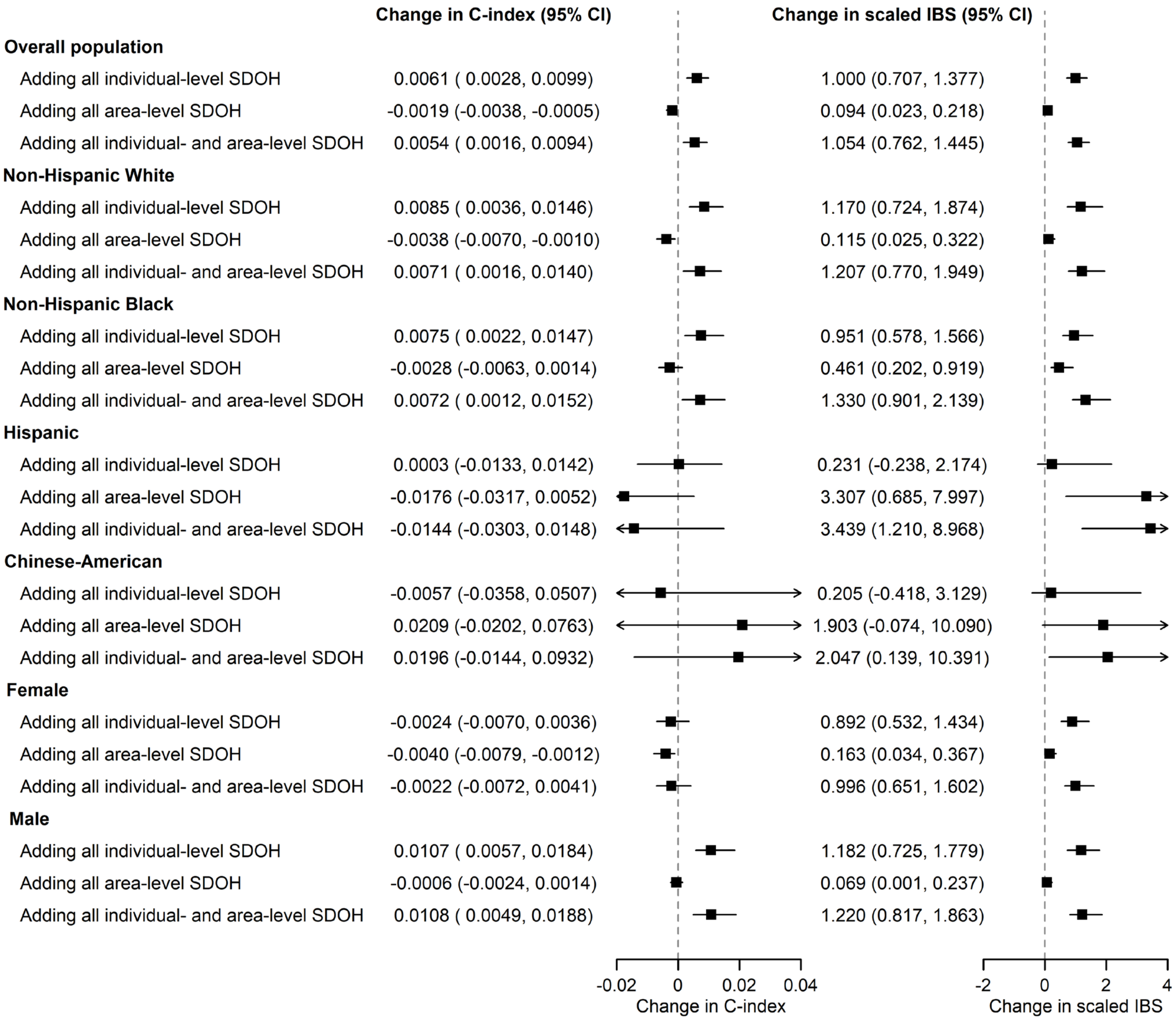

© 2024 Xia M et al. *JAMA Network Open.*

eReferences.

1. Kannel WB, Feinleib M, McNamara PM, Garrison RJ, Castelli WP. An investigation of coronary heart disease in families. The Framingham offspring study. *Am J Epidemiol.* 1979;110(3):281-290.

2. Sempos CT, Bild DE, Manolio TA. Overview of the Jackson Heart Study: a study of cardiovascular diseases in African American men and women. *Am J Med Sci.* 1999;317(3):142-146.

3. Tabb LP, McClure LA, Ortiz A, et al. Assessing the spatial heterogeneity in black-white differences in optimal cardiovascular health and the impact of individual- and neighborhood-level risk factors: The Multi-Ethnic Study of Atherosclerosis (MESA). *Spat Spatiotemporal Epidemiol.* 2020;33:100332.

4. Howard VJ, Cushman M, Pulley L, et al. The reasons for geographic and racial differences in stroke study: objectives and design. *Neuroepidemiology.* 2005;25(3):135-143.

© 2024 Xia M et al. *JAMA Network Open.*
