# Supplementary material for: Cardiovascular Risk Associated With Social Determinants of Health at Individual and Area Levels
Source: JAMA Netw Open. 2024 Apr 26;7(4):e248584. doi: 10.1001/jamanetworkopen.2024.8584 (PMC11053380; doi:10.1001/jamanetworkopen.2024.8584)
Supplement: Supplement 2. — Data Sharing Statement [file jamanetwopen-e248584-s002.pdf]

Data Sharing Statement

Xia. Cardiovascular Risk Associated with Social Determinants of Health at Individual and Area Levels. *JAMA Netw Open*. Published April 26, 2024. doi:10.1001/jamanetworkopen.2024.8584

Data
Data available: No

Additional Information
Explanation for why data not available: Data used in this analyses can be requested from the coordinating center of each cohort studies.
